# Analysis of the current status of "pseudo" unplanned endotracheal extubation in ICU patients in China's tertiary hospitals

**DOI:** 10.1038/s41598-024-64996-4

**Published:** 2024-06-19

**Authors:** Qin Zhang, Li Wang, Xiaoli Liu, Zhenwei Liu, Zaichun Pu, Ziji Fang, Lele Li, Danyang Guo, Rong Lu, Ping Jia

**Affiliations:** 1 Department of Day Surgery, Sichuan Provincial People’s Hospital, School of Medicine, University of Electronic Science and Technology of China, Chengdu, China; 2 Department of NICU, Sichuan Provincial People’s Hospital, School of Medicine, University of Electronic Science and Technology of China, Chengdu, China; 3 Department of Proctology, Deyang People’s Hospital, Deyang, China; 4 School of Medicine, University of Electronic Science and Technology of China, Chengdu, China

**Keywords:** ICU, Unplanned extubation, Pseudo-unplanned extubation, Delayed extubation, Medical-nursing collaboration, Health care, Medical research

## Abstract

To analyze the current status of "pseudo" unplanned endotracheal extubation in ICU patients in China's tertiary hospitals and to provide a reference for improving the quality of medical care. Through the National Nursing Quality Data Platform, unplanned endotracheal extubation data reported by ICUs in China's tertiary hospitals from 2019 to 2022 were analyzed. The situation of reported hospitals, causes, and the current status of "pseudo" unplanned endotracheal extubation in ICU patients was analyzed. The indicator of unplanned endotracheal extubation in ICUs of China’s tertiary hospitals is mainly from first-class tertiary hospitals (74.9%), most of which are self-extractions by patients (74.6%). The proportion of "pseudo" unplanned endotracheal extubation is 45.1%. "Pseudo" unplanned endotracheal extubation is common in the ICUs of China's tertiary hospitals. As such, management blind spots deserve attention from managers and clinical staff.

## Introduction

Unplanned endotracheal extubation (UEE) refers to the endotracheal tube falling out unexpectedly, including patient self-extubation, medical staff inadvertent extubation, and accidental tube slippage^1^. UEE in ICU patients can prolong hospital stays, increase the risk of complications, and even lead to patient death^2,3^. Therefore, its occurrence rate is a critical sensitive indicator of the quality of nursing in ICU patients^4^. However, not all endotracheal tubes need to be reinserted after UEE. The results of a systematic review study showed^5^ that the rate of reinsertion after UEE is about 48.5%, of which the rate of reinsertion after patients self-remove is about 43.8%. This suggests that most patients have reached the criteria for extubation when they self-remove the endotracheal tube, and it is safe to ensure that the tube does not need to be reinserted after extubation. Thus, extubations need to be rethought, whether there is unnecessary delay in extubation for these patients and whether they are unplanned. Based on this, this study proposes the concept of "pseudo" UEE, tentatively defined as ICU patients having reached the criteria for extubation of the endotracheal tube. However, the extubation was not performed or delayed in the ICU patient, and the tube does not need to be reinserted within 24 h after unplanned extubation. Otherwise, it is defined as a "true" unplanned endotracheal extubation. This study aims to understand the current status of "pseudo" UEE in ICUs of tertiary hospitals in China and to propose considerations.

## Materials and methods

### Research subjects

Data was obtained from UEE ICU patients from tertiary hospitals in 23 provinces, 5 autonomous regions, and 4 municipalities directly under the central government of China. Tertiary hospitals mainly refer to Class A tertiary hospitals and Class B tertiary hospitals. The hospital level was determined by the health administrative department of our country according to the evaluation results of the "Hospital Grading Management Standards".

### Data collection methods

The relevant data for UEE reported by the ICUs of tertiary hospitals in Mainland China from 2019 to 2022 were obtained through the National Nursing Quality Data Platform. This included hospital level, total number and cause of UEE, "true" UEE cases (re-intubation within 24 h after UEE occurs), and "false" UEE cases (no re-intubation within 24 h after UEE occurs). The data collection method was to report layer by layer, review level by level, and finally collect it to the National Quality Control Center. The specific collection process was as follows: hospitals at all levels report the above data to the nursing quality control center of the hospital. After being reviewed by the nursing quality control center of the hospital, it was reported to the local municipal quality control center. After being reviewed by the municipal quality control center, it was reported to the provincial quality control center. After being reviewed by the provincial quality control center, it was reported to the national quality control center.

### Observation indicators

Relevant data such as the number and level of hospitals reporting UEE in ICUs of tertiary hospitals each year from 2019 to 2022, the total number of cases of UEE, and the number of "true" and "pseudo" UEE were extracted.

### Data analysis methods

Descriptive analysis was performed using frequency and percentage.

## Results

### The number and distribution of hospitals from which the sensitive indicator “unplanned endotracheal extubation" between 2019 and 2022

Among the tertiary hospitals where the sensitive indicator "Unplanned Endotracheal Extubation" originated, 3358 (74.9%) class A tertiary hospitals and 1123 (25.1%) class B tertiary hospitals were included (Table 1).

**Table 1:** Hospitals that reported the index source of "UEE" from 2019 to 2022.

Hospital level	A particular year				Total
	2019	2020	2021	2022	
Class A tertiary hospitals [n (%)]	838 (78.2%)	756 (75.0%)	874 (74.3%)	890 (72.7%)	3358
Class B tertiary hospitals [n (%)]	233 (21.8%)	252 (25.0%)	303 (25.7%)	335 (27.3%)	1123
Total	1071 (100%)	1008 (100%)	1177 (100%)	1225 (100%)	4481

### Analysis of the causes of UEE in ICUs of tertiary hospitals in our country from 2019 to 2022

See Table 2.

**Table 2:** The causes of UEE in ICUs of tertiary hospitals in China from 2019 to 2022.

Project	A particular year				amount to
	2019	2020	2021	2022	
Patient self-extubation (example) [n (%)]	2075 (74.9%)	1667 (74.8%)	1729 (74.3%)	1491 (74.3%)	6962 (74.6%)
Pipe slip-off (example) [n (%)]	468 (16.9%)	396 (17.8%)	424 (18.3)	353 (17.6%)	1641 (17.6%)
Other reasons (example) [n (%)]	226 (8.2%)	166(7.4%)	172 (7.4%)	163 (8.1%)	727 (7.8%)

### Situations of "true" and "pseudo" UEE in ICUs of tertiary hospitals in China from 2019 to 2022

See Table 3.

**Table 3:** The "true" and "pseudo" UEE in ICUs of tertiary hospitals in China from 2019 to 2022.

Project	A particular year				Total
	2019	2020	2021	2022	
Number of UEE (example) [n (%)]	2769 (100%)	2229 (100%)	2325 (100%)	2007 (100%)	9330 (100%)
Number of "true" UEE (example) [n (%)]	1468 (53.0%)	1186 (53.2%)	1283 (55.2%)	1182 (58.9%)	5119 (54.9%)
Number of "pseudo" UEE (example) [n (%)]	1301 (47.0%)	1043 (46.8%)	1042 (44.8%)	825 (41.1%)	4211 (45.1%)

## Discussion

### Current situation of UEE from 2019 to 2022

Table 3 shows that the reported number of sensitive indicators, such as "Unplanned Endotracheal Extubation," dropped from 2769 cases in 2019 to 2007 in 2022. However, as Table 1 suggests, the number of hospitals reporting this indicator has increased from 1071 to 1225, indicating that the average number of UEE occurrences is decreasing. This suggests that the quality of care, especially critical patient care in our country, is continuously improving, with preliminary achievements in airway management. In recent years, the number of tertiary hospitals has increased annually, with the growth rate of class A tertiary hospitals being higher than that of class B^6^. However, it is noteworthy that while the average number of UEE cases in hospitals is decreasing, the number of hospitals reporting this indicator is increasing. The proportion of reports from Class A tertiary hospitals is decreasing annually, whereas the ratio of reports from Class B tertiary hospitals is increasing. This may suggest that class B tertiary hospitals lack adequate airway management for ICU patients, and the quality of patient care in the ICU has not significantly improved with the overall development of the hospital. Therefore, administrators of class B tertiary hospitals should further strengthen the construction of care quality and standardize airway management based on their resources. Although the total number of UEE cases reported by tertiary hospitals in China was 9330 in the past four years, the number of "pseudo" UEE was 4211, accounting for a high proportion of 45.1%.

### Self-extubation by patients is the main cause of UEE

Data from the National Nursing Quality Platform from 2019 to 2022 shows that self-extubation by patients accounts for most instances of UEE (74.3–74.9%). A systematic review published in 2022 showed that the majority of unplanned extubation of tracheal catheters was done by patients themselves (84.2%), consistent with the conclusion of this study^7^. Research has found that factors such as the patient's illness, delirium, insufficient sedation, improper restraint, delayed extubation, and delayed weaning from mechanical ventilation are all associated risk factors for UEE in ICU patients^8,9^. Furthermore, sedation measures should be discontinued before extubation; a decrease in comfort is also associated with the occurrence of UEE^10^, and all of the above factors can lead to self-extubation by the patient.

### Current state of "pseudo" UEE

Some foreign studies limit the time for re-intubation to within 24 h after extubation^11,12^, and the National Nursing Quality Platform has also adopted this time standard. Therefore, in this study, "pseudo" UEE is also defined as the absence of re-intubation within 24 h following an unplanned extubation. Notably, the analysis revealed that from 2019 to 2022, "pseudo" UEE accounted for 44.1–47.0% of all unplanned extubations, indicating that "pseudo" UEE has been common over the past four years. Moreover, a retrospective analysis published in 1994 found that 52% of UEE patients do not require re-intubation within 24 h and only require safe observation^11^. Another study from Canada found that 57% of children with UEE receive positive pressure non-invasive mechanical ventilation support after a UEE event and do not receive re-intubation within 24 h^13^. A meta-analysis found that 49.8% of UEE patients did not undergo re-intubation, but its re-intubation time was limited to 48 h^7^. It is important to note that “Pseudo” UEE is common in China and other countries. After the patient experienced UEE and did not intubate again under safe conditions, the above patients met the indications for extubation and extubation at the time of the event. Due to certain reasons, the planned extubation has not yet been implemented.

### Unnecessary delay in extubation may be one of the main causes of "pseudo" UEE

Numerous studies^7,11,13^ have found that some patients do not need mechanical ventilation after self-extubation. This study also found that 45.1% of UEE patients were "pseudo" UEE, indicating the possibility of unnecessary prolongation of mechanical ventilation and tracheal tube retention time in the ICU^14^. Although guidelines related to tube extubation exist abroad, there are differences in recommendations. However, there is a lack of current guidelines or expert consensus on extubation for ICU tracheal intubation patients in China^15^, which may cause clinicians to rely mainly on their experience to decide whether a patient is ready for extubation^15^. In our country, physicians lead the removal of endotracheal tubes^15^. However, doctors who cannot be by the patient's bedside continuously may be delayed in grasping dynamic changes in indications for extubation, and making timely and accurate assessments and judgments can be challenging. Thus, clinicians face the challenge of accurately determining the optimal timing for extubation. Moreover, nurses are typically more timely than doctors in evaluating patients' airway self-cleaning ability, peak expiratory flow during coughing, sputum volume, patient grip strength, and airway risk factors before extubation. Furthermore, in clinical work, some healthcare workers might delay the timing of extubation or schedule the procedure when more abundant human or rescue resources are available due to excessive concern for patient safety. Balas MC et al. found that some clinical doctors choose to delay extubation due to lack of confidence in it^16^. However, some scholars believe that for patients with a high risk of tracheal extubation failure, further clinical treatment aimed at improving lung ventilation after spontaneous sreathing trial(SBT) cannot effectively reduce the failure rate of tracheal extubation and delayed extubation increases mechanical ventilation time^17^. In addition, as UEE can cause serious patient harm, managers have intensified management efforts, strengthened existing interventions in many aspects, and added preventive measures. Under scientific management and strict prevention, unnecessary delay in extubation may be overshadowed by the absence of unplanned extubation in patients planned for extubation.

As these patients have already met the criteria for extubation or are in the weaning process, the dosage of sedatives is reduced, and their level of consciousness recovers. As such, keeping the endotracheal tube in place would significantly reduce their comfort levels. A decrease in comfort can increase the risk of patients removing the tube themselves^18^. Therefore, unnecessary delay in extubation may be one of the leading causes of "pseudo" unplanned extubation.

### A nurse-led collaborative model may reduce unnecessary delay in extubation

Medical-nursing collaboration is when doctors and nurses cooperate and make collective decisions to provide patient medical care services^19^. Notably, good medical-nursing collaboration improves medical and nursing levels and promotes patient recovery^20^. Although doctors lead the extubation of patients with endotracheal intubation in our country, nurses play a crucial role in assisting physicians in evaluating extubation criteria, monitoring after extubation, and observing the patients' conditions^17^. On the one hand, planned extubation requires nurses to correctly and timely evaluate, assist in developing individualized extubation plans, fully prepare before extubation, standardize the implementation of extubation, and prevent complications after extubation. On the other hand, an integrated collaboration of medical and nursing staff is needed, particularly in managing sedation and analgesia when UEE occurs. Research^21,22^ has also shown that implementing a nurse-led procedural sedation and analgesia plan can prevent delirium caused by inadequate sedation and analgesia. Delirium is one of the risk factors for UEE (OR = 7.19)^9^. Moreover, other studies have found that a nurse-led programmatic withdrawal plan can significantly shorten the withdrawal time, avoiding unnecessary delayed extubation^23,24^. A pilot RCT study from Spain also demonstrated that a nurse-led tracheal catheter withdrawal plan is feasible and safe^25^. Thus, a nurse-led collaborative care model may reduce unnecessary delays in extubation.

## Limitations and outlook

The literature has no clear concepts associated with "true" or "pseudo" UEE. Therefore, researchers rely on practical definitions. As such, distinguishing between "true" and "pseudo" UEE poses particular difficulties. "Pseudo" UEE is effectively equal to successful extubations in clinical outcomes due to the serious consequences that "true" unplanned extubation can bring to patients. Consequently, "true" and "pseudo" UEE make it difficult to predict clinical consequences when an event occurs. As such, managers may overlook the existence of "pseudo" UEE as a management blind spot to avoid serious clinical harm caused by such adverse events. Therefore, higher standards are required from managers and clinicians. However, the widespread presence of "pseudo" UEE reveals the existence of unnecessary delay in extubation. Therefore, an unnecessary extension of mechanical ventilation time is possible, which is associated with adverse patient outcomes. Although this study suggests that a nurse-led healthcare collaboration model can avoid unnecessary delays in extubation and improve the current situation of "pseudo" UEE, its widespread implementation is challenging due to factors such as China's national conditions, medical system, laws and regulations, and traditional culture and so on. The criterion used in this study for determining "true" or "pseudo" unplanned extubation is whether re-intubation is needed within 24 h after unplanned extubation. However, some scholars propose that the success of extubation can be judged based on whether re-intubation is needed within 48 h after unplanned extubation^26^, a time limit different from this study's criterion. Therefore, more empirical studies are needed to verify these criteria further. Previous studies have found that most clinical doctors believe the current SBT and extubation processes are time-consuming. Moreover, the factors leading to delayed extubation mainly include prolonged SBT time, lack of confidence in implementers' decision-making, and patient-specific factors^17^. However, with the continuous progress of medical diagnosis and treatment technology, the use of advanced medical equipment, especially the widespread use of non-invasive ventilators and high-flow oxygen inhalation after extubation, has reduced the rate of secondary intubation. Therefore, the existing process and plan for tracheal catheter removal can be further optimized to improve the current situation of "pseudo" UEE, which is worth further consideration.

## Data Availability

The data that support the findings of this study are available from National Quality of Care Data Platform, but restrictions apply to the availability of these data, which were used under license for the current study, and so are not publicly available. Data are however available from the authors upon reasonable request and with permission of National Quality of Care Data Platform.
